# Plant–moth community relationships at the degraded urban peat‐bog in Central Europe

**DOI:** 10.1002/ece3.9808

**Published:** 2023-02-13

**Authors:** Mateusz Płóciennik, Krzysztof Pabis, Angelika Zima, Leszek Kucharski, Robert Sobczyk

**Affiliations:** ^1^ Department of Invertebrate Zoology and Hydrobiology University of Lodz Lodz Poland; ^2^ Department of Biogeography, Palaeoecology and Nature Protection University of Lodz Lodz Poland

**Keywords:** moths, nature protection, peatlands, plant communities, plant–moth interaction

## Abstract

Peatlands have their own, specific insect fauna. They are a habitat not only for ubiquistic but also stenotopic moths that feed on plants limited to wet, acid and oligotrophic habitats. In the past, raised bogs and fens were widely distributed in Europe. This has changed since 20th c. Due to irrigation, modern forestry, and increasing human settlement, peatlands have become isolated islands in an agricultural and urban landscape. Here, we analyze the flora in a degraded bog situated in a large Lodz city agglomeration in Poland in relation to the diversity and composition of moth fauna. Over the last 40 years since the bog has become protected as a nature reserve, birch, willow, and alder shrubs replaced the typical raised bog plant communities due to the decreased water level. The analysis of moth communities sampled in 2012 and 2013 indicates dominance of ubiquistic taxa associated with deciduous wetland forests and rushes. Tyrphobiotic and tyrphophile moth taxa were not recorded. We conclude that the absence of moths typical of bog habitats and the dominance of common, woodland species are associated with hydrological changes, the expansion of trees and brushes over typical bog plant communities, and light pollution.

## Graphical abstract

Groundwater level decrease in urban wetlands causes the expansion of common tree and shrub species and the degradation of aquatic flora. This leads to the disappearance of tyrphophile moth species and changes in moth communities that start to resemble typical forest assemblages. These processes are often irreversible, especially in the absence of responsible management from local environment protection agencies.

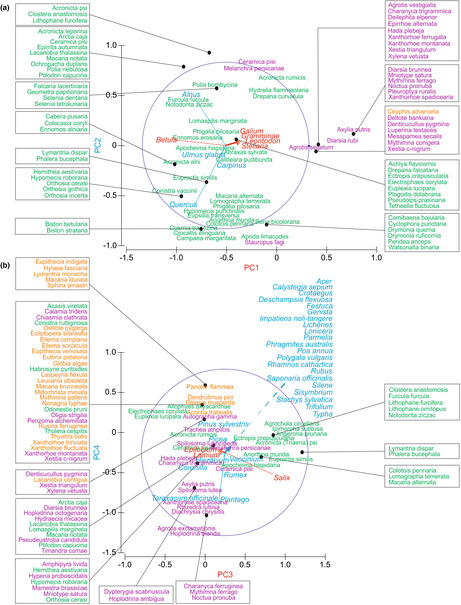

## 1 INTRODUCTION

With more than 160,000 described species, lepidopterans are the largest group of herbivorous invertebrates. They utilize not only the stems and leaves of plants but also their flowers, roots, and seeds (Kristensen et al., 2007). Plant–moth interactions include many other relationships including pollination, and the use of extrafloral nectaries or tree sap (Hahn & Brühl, 2016; Heil, 2015; Majka, 2010; Shrestha et al., 2020). Plants also provide heterogenous and diverse types of habitats for caterpillars and adult insects (Highland et al., 2013; Winiger et al., 2018). Vegetation type may influence not only food availability for caterpillars but also the pressure of predators (Seifert et al., 2015). Moreover, moths and their larvae are an important food source for many birds and mammals, therefore their role in the ecosystems is very important. They are involved in a complex set of mutual interactions with plants and other animals, and constitute a key element of many natural and anthropogenic systems (Nadolski et al., 2021; Singer et al., 2014; Winiger et al., 2018).

Despite high mobility and dispersal potential of many butterflies and moths, their faunas are often strongly related to the structure, composition and/or diversity of local plant communities (Hirao et al., 2008; Shrestha et al., 2020; Thomas, 1996; Truxa & Fiedler, 2012). At the same time, plant–moth diversity relations are still rarely analyzed (Lepš et al., 1998; Root et al., 2017; Tyler, 2020). Those lacks are strongly pronounced in case of endangered habitats, like mires or peatbogs (Spitzer & Danks, 2006). Such wetlands not only serve as refuges for many relict plant communities (Hedwall et al., 2017), but also as habitats for a diverse group of tyrphobionts and tyrphophiles (Spitzer & Danks, 2006). They are among the most threatened European habitats that require special global awareness (Grzybowski & Glińska‐Lewczuk, 2020). In the strongly modified areas of central Europe, those often small‐scale patches of natural vegetation may be considered habitat islands (Bezděk et al., 2006). They are often partially degraded and constitute refuges for rare or specialized species of moths that are absent from neighboring agricultural or urbanized landscape. Such tiny patches of vegetation might constitute small‐scale, local biodiversity hot spots and temporary habitats for good dispersers or species that can be classified as tyrphoneutral (Beck et al., 2002; Bezděk et al., 2006; Infusio & Scalerico, 2018). Our knowledge about the current state of European moth fauna associated with mires and peatbogs is relatively scarce, often limited to basic species lists. There are some studies from Lithuania (Dapkus, 2000, 2004), Poland (Klimczuk & Sielezniew, 2017), Finland (Vӓisӓnen, 1992), and Ireland (Flynn et al., 2016). More extensive research was conducted in the Czech Republic (Bezděk et al., 2006; Jaroš et al., 2014, 2016; Spitzer et al., 1999, 2003; Spitzer & Jaroš, 1993).

The Rąbień Peat Bog can be considered a very interesting and to some extent exceptional area. It has been a nature reserve since 1988 (Kucharski et al., 2004). However, this small (only 42.43 ha) relict mire habitat is located within the borders of a large urbanization complex, between Lodz (one of the largest Polish cities) and neighboring smaller urbanized areas (Kucharski et al., 2004; Mamiński, 1987). Its role in the strongly fragmented and degraded urbanized landscape is therefore also crucial from the point of view of local biodiversity preservation, since its fauna and flora include not only typical mire species, but also a variety of opportunistic taxa associated with urbanized or agricultural areas. Moth communities are considered good indicators of ecosystem response to changing environmental conditions and might be used for monitoring future changes in insect and plant communities at a given location (An & Choi, 2013; Kitching et al., 2000; Rákosy & Schmitt, 2011). Therefore, the aim of our study was to consider the relationships between plant and moth communities at this endangered wetland and discuss some future scenarios of changes in this small nature reserve. The data about the current moth fauna sampled in 2012 and 2013 were discussed against a background of available long‐term temporal data describing changes in plant communities. We hypothesize that degradation of peat bog flora will be reflected in the composition of the current moth communities in the studied area. This process can have three aspects:

1. moths are mobile insects, which is why there may be no distinct differences in adults composition between more and less degraded areas of the peatland;
2. nevertheless these taxa, in a caterpillar stage, are associated with host plants that grow in different areas in the local landscape depending on the condition of the bog;
3. thyrphophile and herb host plant‐related moths may be displaced over time in the degraded peatlands by moths associated with deciduous trees and shrubs following hydrological changes and forest expansion.

## 2 MATERIALS AND METHODS

### 2.1 Study area—Present and past plant communities

Human activities have a direct impact on the transformation of vegetation. Its measure is the difference between the real and potential community for the analyzed habitat (Kostrowicki et al., 1988). Strong pressure caused by the increasing urbanization of areas directly adjacent to the “Torfowisko Rąbień” reserve causes major changes in its habitat and the vegetation growing in it.

**FIGURE 1:**
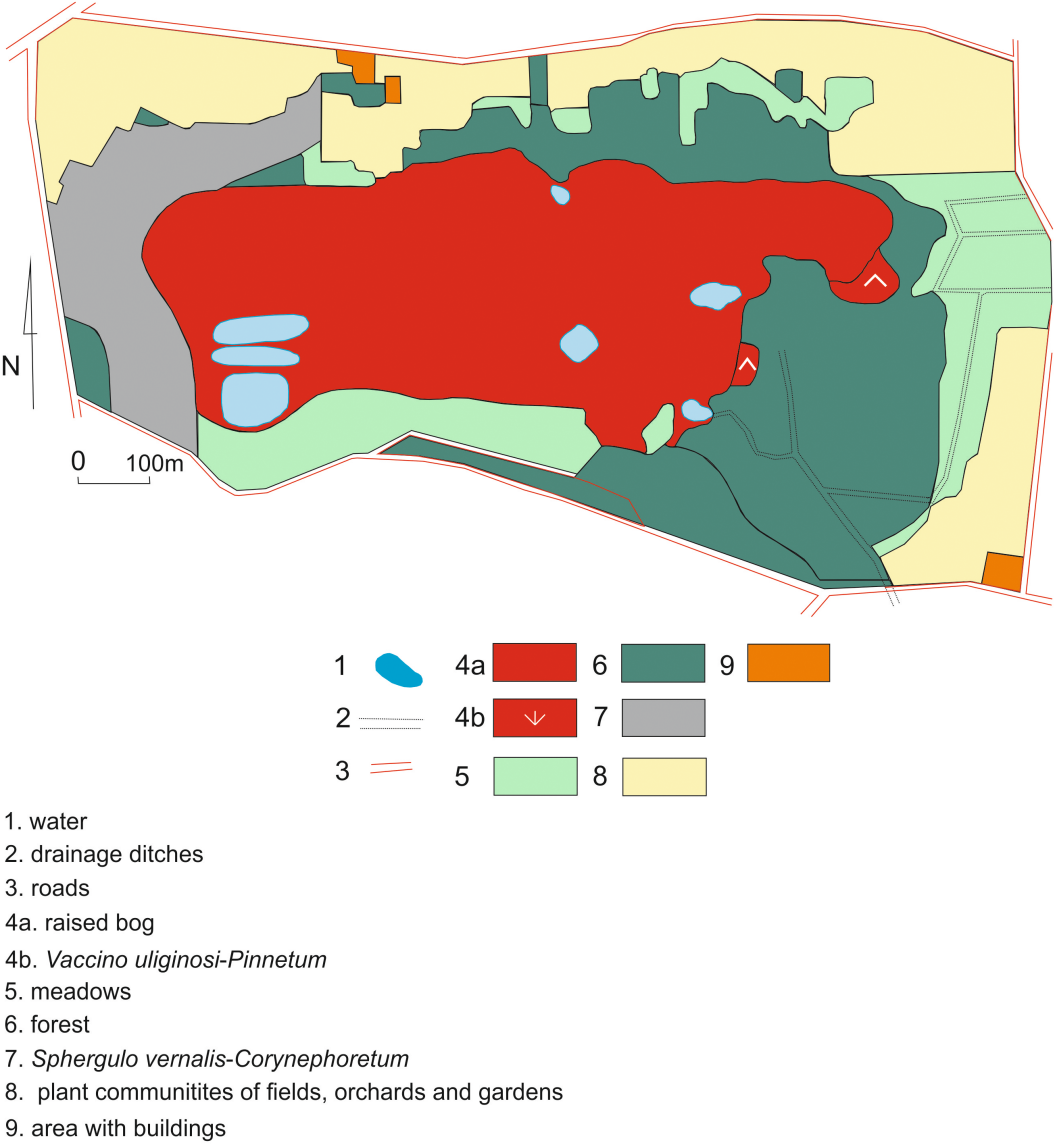
Vegetation of the “Torfowisko Rąbień” reserve and its surroundings in the 1980s (by Mamiński, 1987). 1—water; 2—drainage ditches; 3—roads; 4a —complex of communities: *Eriophorum vaginatum‐Sphagnum fallax* community, *Eriophoro angustifolii‐Sphagnetum recurvi*, *Phragmition*; 4b ‐ *Vaccinio* *uliginosi*‐*Pinetum*; 5—meadow community of *Molinio‐Arrhenatheretea* class; 6—*Sphagno squarrosi‐Alnetum, Querco roboris‐Pinetum*; 7 ‐*Spergulo* *vernalis*‐*Corynephoretum* and young pine forest; 8— plant communities of fields, orchards and gardens; 9—areas with building.

The mire in Rąbień near Łódź is located south of a compact range of lowland bogs (Tobolski, 2003). Research on bog vegetation carried out at the turn of the 1980s and 1990s showed that almost half of the object's surface was covered by vegetation typical of raised bogs (Mamiński, 1987; Figure 1). It was dominated by a mosaic of communities typical of the *Oxycocco‐Sphagnetea* raised bogs and the *Scheuchzerio‐Caricetea nigrae* transitional bogs. *Vaccinio uliginosi‐Pinetum* swamp forest covered a small part of the bog's surface. Vegetation was characterized by a large share of bryophytes, mainly *Sphagnum* sp. The vascular flora was dominated by the sedges *Carex* sp. and *Eriophorum angustifolium* as well as shrubs and trees (*Betula* sp. and *Pinus sylvestris*). Peat pits were overgrown by *Phragmites australis*, *Typha latifolia*, and high sedges. A garbage dump was located in the south‐western part of the depleted bog. It functioned here in the 1960s. In the first period, after cessation of waste storage, annual and biennial plants dominated there. Vegetation here consisted of species of the genera *Chenopodium*, *Sisymbrium*, and *Atriplex*. In subsequent years, they gave way to perennial plants. The area of the community increased, with tansy dominating *Artemisio‐Tanacetetum vulgare*, in which, apart from the dominant, *Urtica dioica*, *Artemisia vulgaris*, and *Arctium lappa* were noted.

There were also small areas of grassland from *Plantaginetalia* formed by: low grasses, *Plantago major*, and *Polygonum* sp. From the south and partly from the east, the bog was surrounded by pine forests with a small admixture of *Quercus robur*. The areas adjacent to the site in the north and east (swampy part) were overgrown by alder carr (*Sphagno squarrosi‐Alnetum* and the *Betula pendula* community). Settlement entered the areas adjacent to the reserve from the north, east, and south. In the west, the bog borders on a dune, which was largely covered by the *Spergulo vernalis‐Corynephoretum* and self‐seeding *Pinus sylvestris*.

**FIGURE 2:**
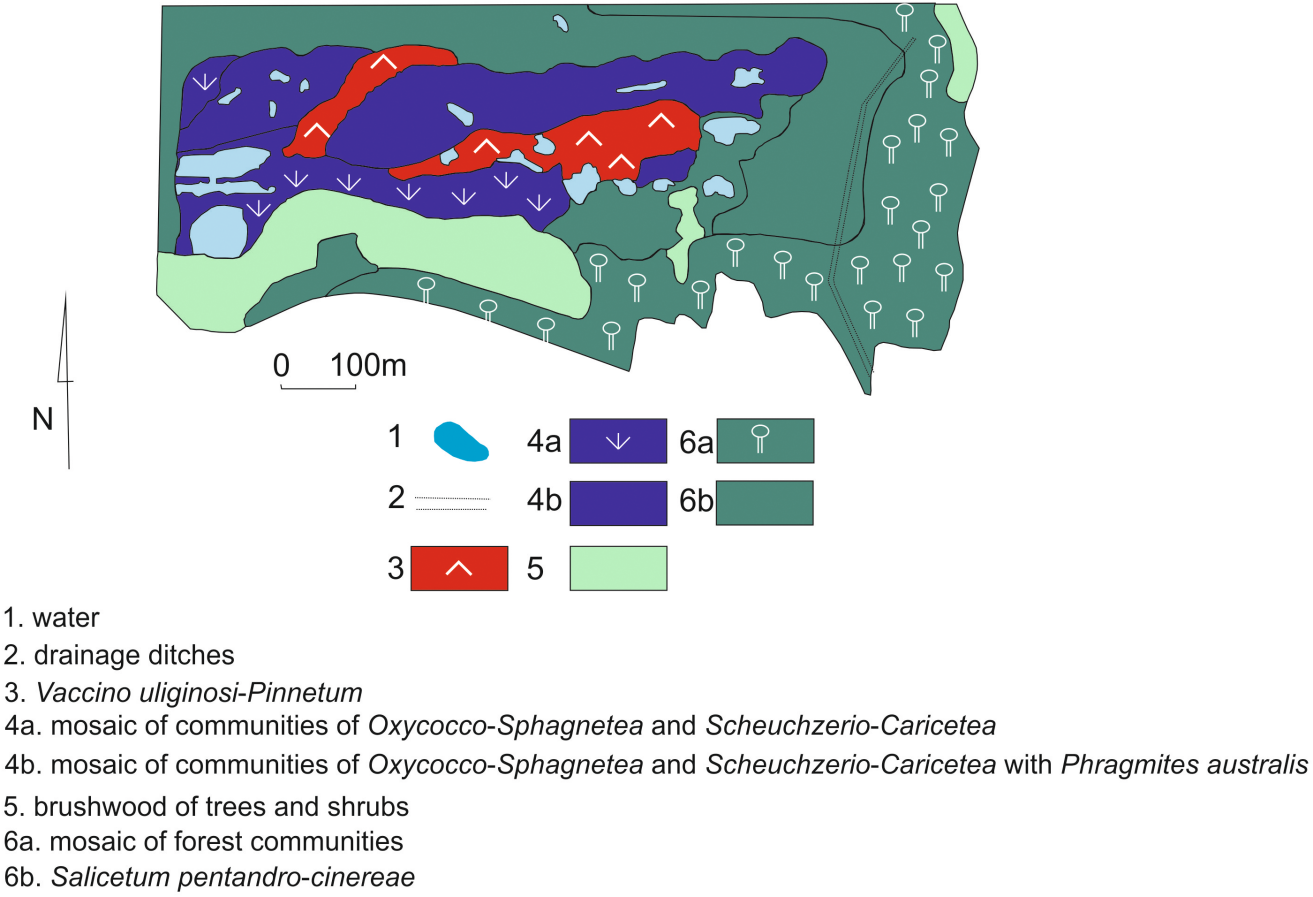
Vegetation of the “Torfowisko Rąbień” reserve in 2000. 1—water; 2—drainage ditch; 3—*Vaccinio uliginosi‐Pinetum*; 4a—complex of communities: *Eriophorum vaginatum‐Sphagnum fallax* community, *Eriophoro angustifolii‐Sphagnetum recurvi*, *Phragmition*; 4b—complex of communities: *Eriophorum* *vaginatum*‐*Sphagnum* *fallax* community, *Eriophoro* *angustifolii*‐*Sphagnetum* *recurvi*, *Phragmition* with *Phragmites* *australis*; 5—brushwood of trees and shurbs‐; 6a—*Querco* *roboris*‐*Pinetum* ; 6b—*Salicetum* *pentandro*‐*cinereae*.

Vegetation tests repeated in the years 1999–2000 showed changes occurring in it. They included an increase in participation of young trees (*Betula pendula*, *B. pubescens*, and *Pinus sylvestris*) and *Salix cinerea* in peat bog vegetation (Kucharski et al., 2004). As a result, the forest area increased (Figure 2). The area of the *Phragmites australis* community has also increased. An indicator of changes occurring in the vegetation was the disappearance of species characteristic of oligotrophic habitats, which were replaced by taxa typical meso‐ and eutrophic waters and soils (Kucharski et al., 2004; Sitkowska, 1996). Mosaic vegetation increased in the central part of the bog, which was the result of succession processes (Figure 2). Anthropogenic vegetation in the south‐western part of the reserve underwent reconstruction. It was overwhelmed by a collection of tall perennials, including *Tanacetum vulgare*, *Galium aparine*, *Arctium lappa*, and *Artemisia vulgaris*. The first tree seedlings appeared in it (including *Acer negundo*). The forest area surrounding the interior of the peat bog increased at the expense of non‐forest peat vegetation. The areas directly adjacent to the reserve from the north, east, and south have changed further because of urbanization. The dune stretching along the western border of the reserve was overgrown by a young pine forest.

**FIGURE 3:**
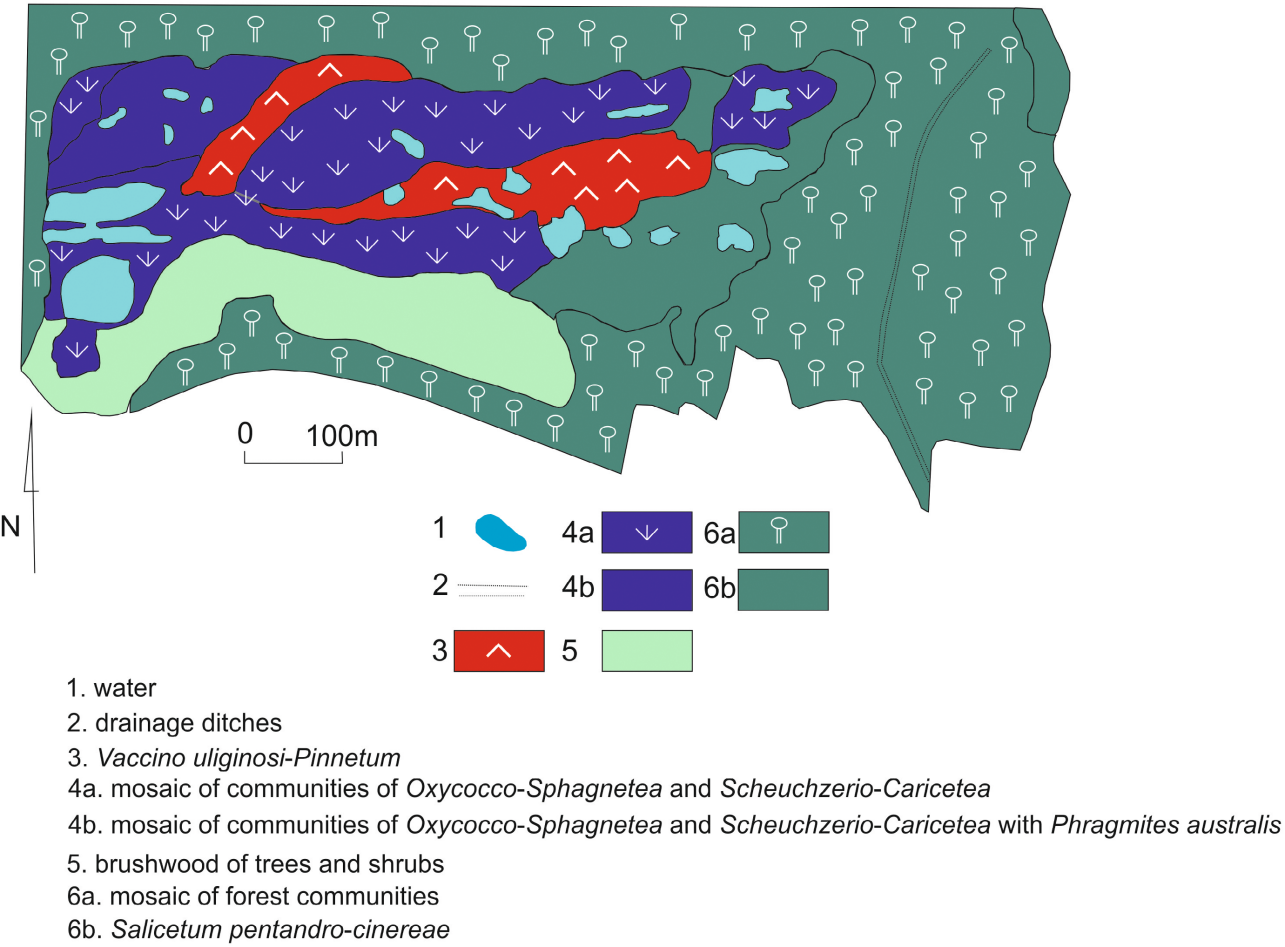
Vegetation of the “Torfowisko Rąbień” reserve in 2018. 1 ‐ water; 2 ‐ drainage ditches; 3 ‐ *Vaccinio* *uliginosi*‐*Pinetum*; 4a ‐ mosaic of communities of *Oxycocco*‐*Sphagnetea* and *Scheuchzerio*‐*Caricetea* *fuscae*; 4b ‐ mosaic of communities of *Oxycocco*‐*Sphagnetea* and *Scheuchzerio*‐*Caricetea* *fuscae* with *Phragmites australi* *s*; 5 ‐ brushwood of trees and shurbs; 6a ‐ mosaic of forest communities, mainly *Querco roboris*‐*Pinetum*; 6b ‐ *Salicetum* *pentandro*‐*cinereae*.

The inspection of the reserve carried out in 2018 confirmed a significant increase in the area of forest and scrub vegetation at the expense of peat bog (Figure 3). *Phragmites australis* has captured almost ¾ of the area previously occupied by a mosaic of communities of *Oxycocco‐Sphagnetea* and *Scheuchzerio‐Caricetea fuscae*. The area of *Phragmitetum australis* increased by 100% compared to the previous state. The area of the former garbage dump is currently occupied by a mosaic of thickets of trees and shrubs and patches of *Artemisio‐Tanacetetum vulgare*. However, the surface of *Plantaginetalia* grasslands did not change. The buildings of the town surrounding the peat bog are now directly adjacent to the reserve borders from the north and east. On the west side, they are separated by a pine forest dune, and in the south by a *Quercus robur* forest.

Its specific location means that it belongs to the endangered elements of the lowland Poland landscape. Its location and various types of soil structure disorders contribute to habitat changes and the spread of synanthropic plants. These factors are the main reasons for the reconstruction of the vegetation of the characterized object. They can significantly affect not only its flora, but also the fauna occurring in this area.

### 2.2 Sampling methods and material identification

Moths were caught using light traps, as this method is considered reliable in studies of moth composition in relation to habitat. Earlier studies demonstrated that the flight distance is relatively small, and generally light traps attract moths mostly from the local habitat near to the source of the light (Baker & Sadovy, 1978; Beck & Linsenmair, 2006; Truxa & Fiedler, 2012).

The material was collected from spring of 2011 to late autumn of 2012. At the Różana 30B street (51°48′00″N; 19°18′42″; R) site, a Robinson trap with mercury bulb was used, and sampling took place from spring 2011 to spring 2012. Catching was carried out regularly at two‐week intervals from 9:00 pm to 3:00 am. The catch at Torfowa Street (T; 51°48′08″N; 19°17′38″) took place by a standard light trap with mercury‐incandescent bulb and white canvas from spring to autumn 2012 at 2‐week intervals. The light trap at Torfowa st. took place between 10.00 pm and 12.00 pm. The data matrix presenting a list of taxa collected at R and T sites is included in appendices, in Tables S1 and S2. The Heterocera were poisoned with ethyl acetate and then frozen until preparation. The habitat and host plant preferences of collected moth species were obtained from literature data, especially those focused on central European fauna (e.g., Buszko & Masłowski, 2012; Nowacki, 1998; Waring & Townsend, 2009). Such approach to functional or ecological classification of animal communities is used in other insect studies (e.g., Muto‐Fujita et al., 2017; Strutzenberger & Fiedler, 2022) but also in research focused on other groups of invertebrates, and allows for multivariate analysis and modeling (Sobczyk et al., 2021). This allows for a comprehensive classification based on detailed multisource knowledge about biology and ecology of particular species. Taxonomic nomenclature was adopted based on lepidoptera.eu (accessed at 2022). The reference collection is deposited in the Department of Invertebrate Zoology and Hydrobiology, University of Lodz and in a personal collection of Angelika Zima. Permission for research in the vicinity of the reserve was issued by the Regional Directorate for Environmental Protection in Lodz.

### 2.3 Statistical analysis

To identify habitat preferences and main host plants of the species collected, multivariate methods were used—Non‐metric MultiDimensional Scaling (NMDS), SIMilarity PERcentage (SIMPER), and Principal Component Analysis (PCA). NMDS is a very good and widely used method for analysis of similarity, both for abundance and presence/absence data. It allows to find the patterns in community composition or ecological similarity between species groups. SIMPER and NMDS are two complementary methods. SIMPER allows for a more detailed insight into the community composition and selection of the most important taxa responsible for the observed pattern. Principal Component Analysis (PCA) was used to identify how host plants explain the variability in moth communities. As the PRIMER 6 was used, the eigenvectors identify well each host plant (as an environmental variables) correlation to the PC Axes and Principal Component Scores the location of moth taxa (as samples) at the multivariate space (Clarke & Gorley, 2015).

- First, SIMPER analysis was conducted to find the main habitats preferred by taxa collected at Tofrowa (T) and Różana (R) sites, and dissimilarities between habitat preferences of taxa reported at R and T sites. For this propose, moth species were classified as “samples” and divided into three groups—(1) collected only at T site, (2) collected only at R site, (3) collected at both sites. The main preferred habitats (up to three) of each moth taxa were classified as “variables,” and one‐way SIMPER analysis using Bray–Curtis similarity and cut‐off of 100% was conducted to define their most preferred habitats.
- To recognize an assemblage pattern in caterpillar host plants of the reported taxa, a series of analysis was used. (1) NMDS analysis based on Bray–Curtis formula was conducted on all collected moth species as “samples,” and for each species up to three main host plants as “variables.” As NMDS divided taxa into two main moth groups—A and B, and an out‐group O, (2) one‐way SIMPER analysis indicating main host plants for each of these groups was conducted using Bray–Curtis similarity index and 100% cut‐off. (3) For summarizing the pattern of the moth–host plant relations in the nature reserve, PCA was conducted regarding moth species as “samples” and host plants as “variables.” It presents the most important host plants, and the main moth taxa groups related to their occurrence.

All statistics were performed with PRIMER 6 (Clarke & Gorley, 2015) and Past (Hammer et al., 2001) software.

## 3 RESULTS

### 3.1 Moth communities—Habitats and host plants

The SIMPER analysis indicates that the main preferred habitats of species collected at the R and T sites are more or less the same, and their average dissimilarity is 73.58% (for R & T site catch). These are wetland alder forests (40.81% and 53.55%, respectively), meadows and other open grass and herb habitats (17.72% and 19.44%), gardens (13.98% and 10.69%), clumps of trees in the cultural landscape (10.2% and 6.67%). The only difference is that at the R site, there is a 9.67% share of species preferring oak deciduous forests, whereas at the T site (6.27%), there are taxa typical of pine coniferous forests. The difference follows the character of the areas neighboring the light trap plant communities—at R, these are gardens and old mixed forests with oaks and birches, while at T, these are young pine plantations and meadows. For both sites, there are common species preferring aquatic habitats (3.09%) which are in the direct vicinity of T but not R study site.

**FIGURE 4:**
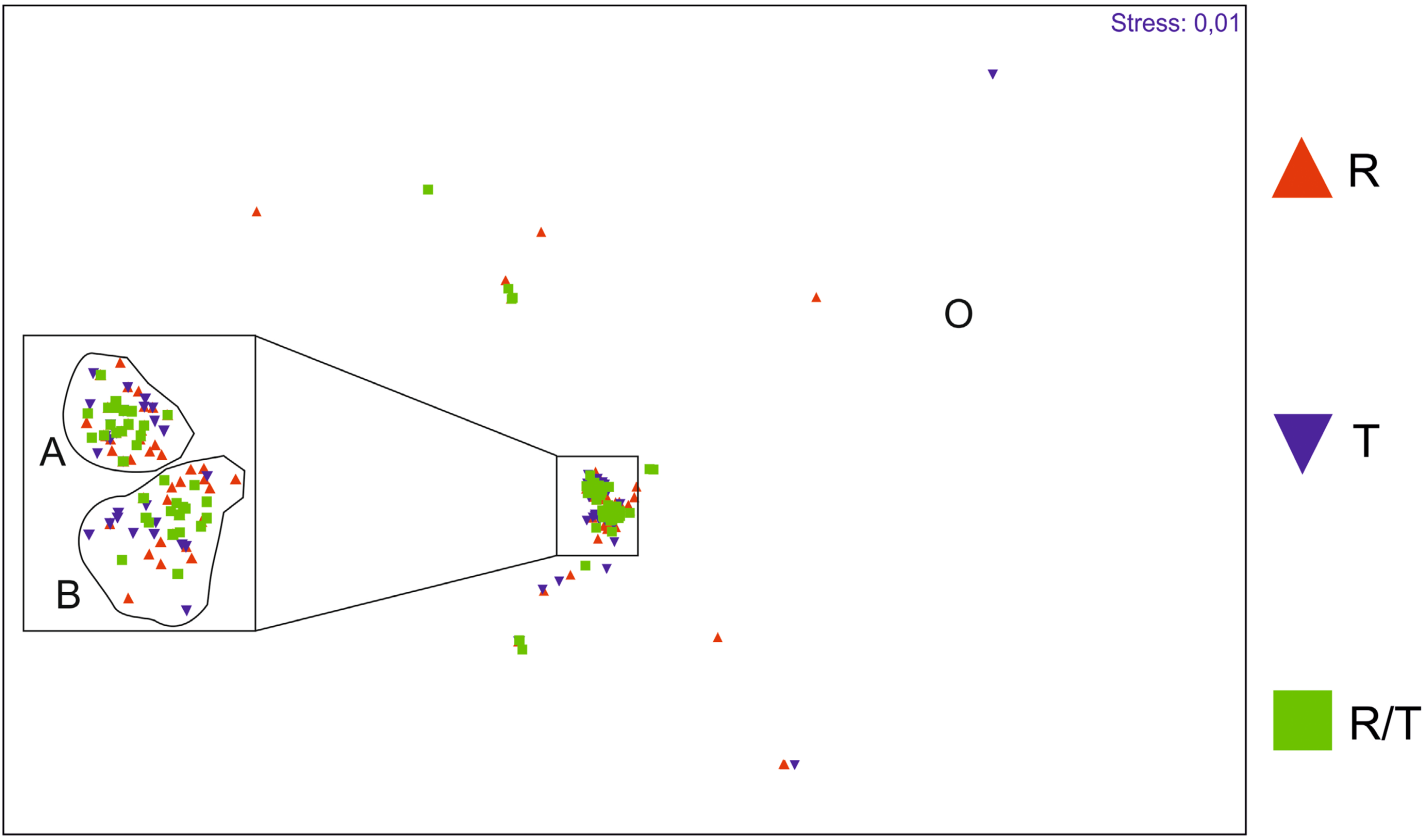
Plot illustrating NMDS analysis. Similarity of caterpillar host plant preferences of moth species collected only at Różana site (R), only at Torfowa site (T), at both sites (R/T). Groups A, B, and O are marked.

**FIGURE 5:**
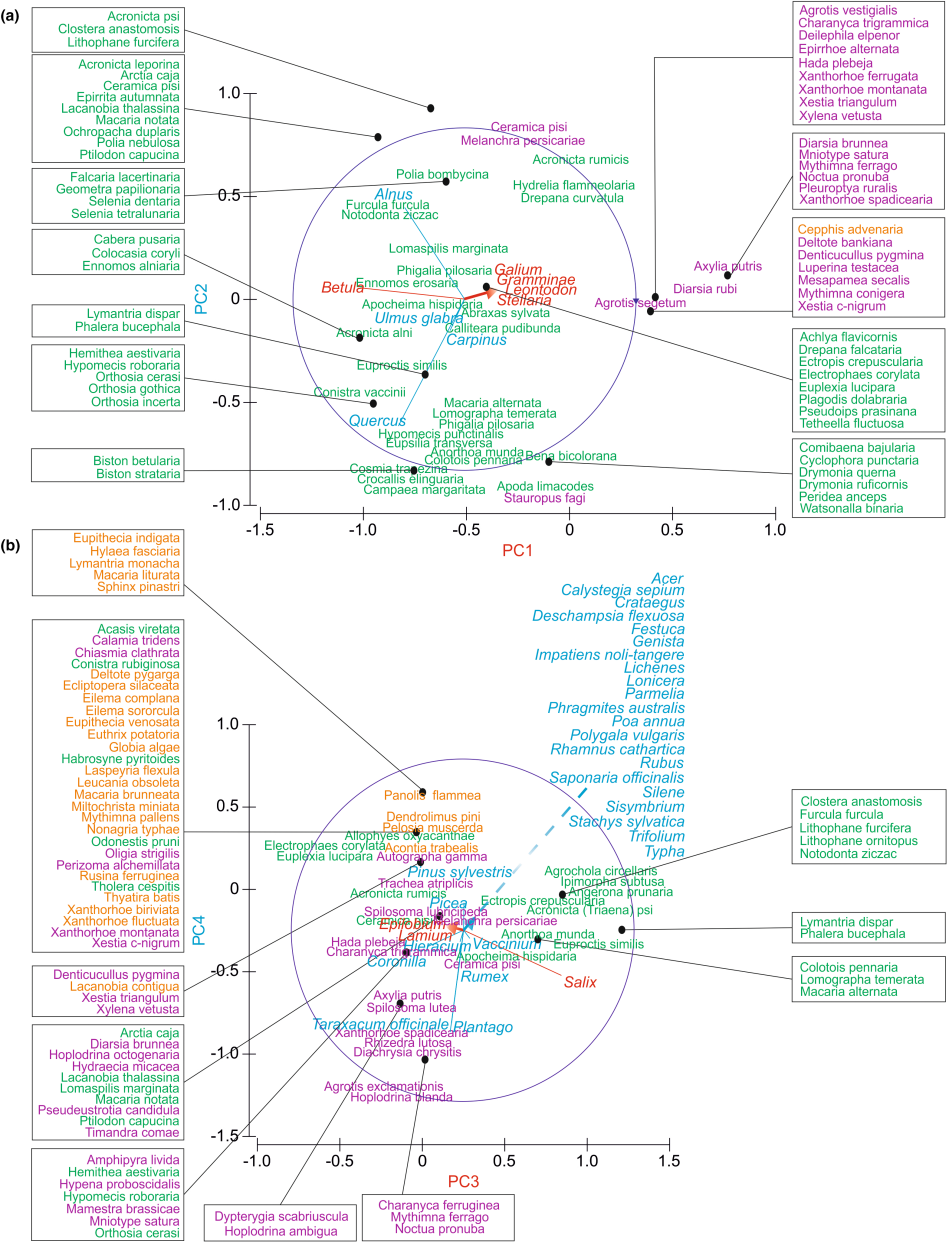
Results of PCA. (a) Plot illustrating PC1 and PC2, (b) plot illustrating PC3 and PC4. Red lines and axes illustrate host plants correlated at plot (a)—with PC1, plot (b)—with PC3; blue lines and axes illustrate host plants correlated at plot (a)—with PC2, plot (b)—with PC4. Colors of moth names illustrate: green—taxa in group A associated with deciduous trees, violet—taxa in group B associated with herbs, orange—taxa in group O associated with other host plants, including coniferous trees. Plot (a) only includes color‐coded names of those correlated with PC1 and PC2, plot (b) includes only those correlated with PC3 and PC4. Taxa not corrected with PC1 and PC2, nor not correlated with PC3 and PC4 are not illustrated.

As mentioned above, the NMDS analysis (Figure 4) divided the collected species into two groups, A and B, as well as an outgroup O. The NMDS indicates that species from A, B, and O groups were collected at both sites T and R. Nonetheless, SIMPER proves they are well separated (Table S3). Group A includes species, whose caterpillars feed on deciduous trees: birch (36.22%), oak (23.56%), poplar (13.61%), willow (12.19%), and alder (11.03%). This group is well specified, having an average similarity of 30.84%. It indicates that, whereas alder wetland forests are the most popular habitats for moths, *Alnus* sp. and *Salix* spp. are less preferred as host plants than deciduous trees typical of more dry habitats. Group B includes taxa feeding mainly on herbs and grasses—sorrel (18.92%), plantain (18.92%), dandelion (12.90%), nettle (12.77%), grasses (11.69%), bedstraw (7.70%), pigweed (4.48%), and chickweed (3.87%). This group is less specific than A, having an average similarity of only 12.52%. Other taxa not aggregated in A or B were classified as an outgroup and it is not surprising that group O has the lowest average similarity of 7.93% (Figure 4). It aggregates moth species with diverse host plants in larval stage—coniferous trees—pine (33.33%) and spruce (21.74%), lichens (23.19%), but also honeysuckle (6.76%), which is not reported from the reserve but possibly cultivated at the local gardens, and catchfly (5.31%) common in open habitats and near ground roads. All three groups are very distinct, having an average dissimilarity ranging from 98.61% (A & B groups) to 99.97% (A & O). The list of moth taxa belonging to each host plant group (A, B, O) is presented in Table S4, and the data matrices presenting the main habitat and host plant preferences of the collected species are included in Table S1 and Table S2. Additionally, PCA reveals the ordination of all collected taxa in the host plant community space. The PC1 axis explains 17.1%, PC2–8.8%, PC3–8.4%, and PC4–6.5% of the moth–host plant relation variability (Figure 5a,b). The omitted PC5 reflects 5.7% of variability. PC1 distinguishes groups A and B, indicating birch as the most important host plant designating group A, and bedstraw, grasses, hawkbits, and starwort as the most important taxa designating group B. Oak, hornbeam, elm, and alder are trees that differentiate the taxa from group A into two subgroups—more related to alder wetland forests and typical of drier deciduous forests. PC3 reveals willow as a second tree taxa designating another subgroup of A, as well as willowherb with dead‐nettles as distinguishing second subgroup of B. PC4 generally separates those taxa from group O, which feed on pine and spruce from the taxa in group B, that is, those feeding on plantain, common dandelion, sorrel, *Coronilla*, hawkweed, and *Vaccinium* berries.

## 4 DISCUSSION

### 4.1 Past changes in plant communities as an implication for current distribution of moth trophic groups

Since available temporal data about changes in plant communities are being confronted with data on moth fauna from two seasons only (present state of fauna) we can only extrapolate the currently observed patterns to possible scenarios of changes in the studied area. Based on our results, we hypothesize that, as trees expanded in the reserve area from the 1980s to the present time (Mamiński, 1987; Kucharski et al., 2004; botanical survey in 2018 presented in detais above (Section 2.1)), group A of moth taxa increase in its abundance and range in the wetland. It is visible in the composition of the current fauna, as group A is the most speciose (83 species) and dominates the current moth community. This concerns the taxa associated with most important host trees—birch, willow, and in less extend alders and oaks. The willow‐associated moth subgroup of cluster A probably expanded later when it precipitated succession of the central part of raised bog patches. We can assume that, at present, the willow‐associated moth group is a major part of the fauna in the reserve, as *Salicetum pentandro‐cinereae* occupy most of open habitats in the Rąbień wetland. Although further quantitative studies are needed to fully answer this question. The presence of alder‐associated moth group of cluster A is related to wet fragments of the reserve, where young brushwood and brushes appear. However, alder trees surrounded bog patches from the beginning of the investigated period of Rąbień vegetation history and it is probable that those taxa were constant elements of the community also in the past. Small patches of alder bush are also currently present at the north‐east margins of the reserve. In the past, this part of wetland was covered by a meadow (Mamiński, 1987). After it was abandoned, it was quickly colonized by *Alnus glutinosa* (Kucharski et al., 2004). Whereas this species has been present at the margins of the reserve for a long time, if the process of soil eutrophication does not stop, alder and birch may colonize all wetland. As groundwater levels will continue to decrease because of human settlement surrounding the wetland, in the next few dozen years, *Sphagno squarrosi‐Alnetum* plant communities might cover the whole reserve. Deciduous and mixed forests have occupied the eastern part of the reserve for a long time, and are natural in character. Birch expanded quickly in the wetland at the first stage of the degradation, before year 2000 (Kucharski et al., 2004). Now, it still spread on areas where water levels are decreasing. This is the most important tree host taxa in moth group A. The oak‐associated moth group of cluster A should be considered as a stable component of moth communities in eastern, dryer part of the reserve. The oak and hornbeam are not clearly expanding in the reserve. As the most important species for group O, pine is a stable component of coniferous forests in eastern part of the reserve and its southern vicinity in mixed oak‐birch‐pine, and coniferous pine‐spruce plantations. The expansion of *Pinus* started on the dune in the eastern margins of the reserve and in the central part of the bog composing patches of *Vacinio‐uliginosi Pinetum* even before 2000 (Kucharski et al., 2004). Now, the pine population in the reserve is more stable, and its expansion in the wetland is not as quick as deciduous shrubs and trees. It suggests that moth group O expanded in the wetland mostly before 2000.

Within moth group B, there are three subgroups of moths related to herbaceous host plants of non‐forest communities. Group B is still quite speciose, although it consists of 57 species and is therefore much less diverse than a tree‐related group. It might be associated with an expansion of shrubs and trees. The first subgroup of moth taxa B is related to host plants like *Galium*, *Stellaria*, *Leontodon*, and some species of grasses. They grow in open wetland areas, water body margins, and damp meadows. In the “Rąbień Bog” reserve these habitats are present in the western and central areas, where there are patches of peatland vegetation (*Scheuchzerio‐Caricetea nigrae* and *Oxycocco‐Sphagnetea*) with pits and ponds created after peat extraction. From the 1980s to 2018, the surface area of these habitats decreased because of the expansion of reeds and trees (Mamiński, 1987; botanical survey in 2018). Some of the host plants from *Galium‐Stellaria*‐associated moth subgroup appear in substitute habitats in rushes and wet leafy forest. Moth species related to *Galium‐Stellaria* host plants are currently a stable component of reserve fauna. The next subgroup is *Epilobium‐Lamium*‐associated moth taxa. During the 1960s, the southern part of the Rąbień peatland was partially changed into a waste dump, which resulted in a ruderal plants colonization (Mamiński, 1987). At first, that were annuals, then perennials. During the 1990s, this area was dominated by a plant community with *Tanacetum vulgare* and *Artemisia vulgaris*. This community includes many species from the *Epilobium‐Lamium*' host plants. The succession of shrubs and trees in these habitats in the 21st century caused the regression of *Epilobium‐Lamium* host plants, especially next to sampling site T (Kucharski et al., 2004; botanical survey in 2018). The last moth subgroup, B, is composed of moth taxa feeding mainly on *Plantago, Rumex, Vaccinium, Hieracium*, *Taraxacum officinale*, and *Coronilla varia*. These host plants are related to dirt paths and ground roads. They often appear on dry, warm swards and in pine forests (Buszko & Masłowski, 2012; Nowacki, 1998; Sterling et al., 2020). The area of swards has not changed during the last 40 years. They occupy a small but stable area in the Rąbień wetland. In the pine forests, those host plants are gradually being replaced by *Vaccinium*.

As a result of all of the above‐mentioned processes, the primary character of the Rąbień peat bog changed into a mosaic of different habitats, also resulting in high ecofuntional diversity of moth fauna including the species related to different types of plant species and microhabitats. High diversity is often considered a value in itself, although in case of the peatbog habitats, it points at substantial degradation and undesirable character of the current moth communities. The current state of the moth fauna based on our data can be considered a benchmark knowledge for studies of future changes in this reserve or potential recovery actions.

### 4.2 Protection of Rąbień nature reserve and suggestions for its future management

The degraded character of plant communities in the Rąbień peat bog is reflected in the composition of moth fauna. Our results strongly contrast with the observations from typical central European mires and peatbogs, where the number of moths characteristic for this type of habitat oscillated around 30–50 species with at least several species of tyrphobionts (e.g., Dapkus, 2000; Spitzer & Jaroš, 1993, 2014). There was an almost complete lack of tyrphobiontic and tyrphophilous taxa in our study. Only geometrid moth *Pennithera fermata* belongs to less common moths that are associated with peatbogs, although this species was observed in the whole Poland and can also be found in pine forests (Buszko & Nowacki, 2017). Rabień is also a habitat of rare and local *Drymonia querna*, although this species was already recorded in other nature reserves located in the vicinity of Lodz agglomeration and is not associated with wetlands (Pabis, 2003). We also did not record any species of aquatic moths that are often found in such habitats (Pabis, 2018). The only exception is a common semiaquatic noctuid *Nonagria typhae* associated with *Typha* (Nowacki, 1998). The absence of bog‐associated species was to some point surprising, as many typical peat bog plants are still abundant in the reserve. During the studies of moth fauna associated with degraded peat bogs in Ireland, tyrhobiontic species were recorded even at disturbed sites (Flynn et al., 2016). Our results are therefore most probably associated with very strong influence of urbanization and other types of human‐related pressure in the areas neighboring the Rabień Bog.

Despite the lack of typical tyrphobiontic taxa, Rabień might be considered a habitat refuge for the local moth population. Our results demonstrated strong relation between the composition of plant communities and the composition of moth fauna. Rabień is an excellent example of succession changes and anthropopressure influence within a relict ecosystem. Increasing urbanization in the vicinity of the “Rąbień Bog” reserve is one of the main factors leading to groundwater level decrease. This caused peat decomposition and soil eutrophication, influencing local algae flora and replacing plant taxa related to oligotrophic conditions with those preferring meso‐ and eutrophic edaphic conditions (Kucharski et al., 2004; Sitkowska, 1996). The next ongoing phenomenon is the expansion of *Phragmites australis* (Figures 2 and 3), followed by the spread of rushes and forest communities. The degradation that led to substantial changes in plant communities most probably resulted in strong shifts in the composition of the moth fauna. Nevertheless, there are no historic data about moths associated with this area. Based on the data collected in the 1960s and 1970s during other faunistic studies from neighboring areas, we can assume that at least some tyrphobitoic species were also found in Rąbień. Many of those species are already extinct in other, much less disturbed peat bogs in central Poland (Marciniak & Śliwiński, 1988; Śliwiński, 1960, 1995a, 1995b, 1995c, 1996a, 1996b, 1998; Śliwiński & Marciniak, 1991).

On the other hand, our results demonstrated that the role of this area as a nature reserve is still important, especially as a habitat island located in a strongly urbanized area. Jaroš et al. (2014) postulated a great need for restoration of such relict habitats in central Europe. Boreal peat bogs are one of the most threatened habitats in this part of the world (Spitzer & Danks, 2006). Soumarsky Mos bog is a good example that such restoration is possible even in post‐harvested peat bog, and that recolonization of relict insects is still possible (Jaroš et al., 2014). Rąbień was not destroyed by harvesting, although it suffers from strong urbanization pressure and requires restoration of hydrologic conditions that might be very difficult to achieve due to the specific location of this nature reserve. Studies show, that the regeneration of vegetation after human‐induced disturbance might be slower than after natural changes (Bastl et al., 2009). However, recent observations from Canada have shown that the restoration and rewetting of urban peat bogs might be successful (Christen et al., 2016). At the same time, Rąbień might be a great place for various citizen science‐based projects, where people living close to this reserve might be involved in the monitoring and restoration activities (Colston et al., 2015). Rąbień certainly needs a good management strategy that will allow for the restoration and monitoring of plant and animal communities. Without those urgent activities, the degradation of this peat bog will proceed, leading to complete loss of its role as local small‐scale urban biodiversity hot spot, probably much more efficient than typical urban parks (Nielsen et al., 2013; Śliwiński & Marciniak, 1991). Our results demonstrated relatively high diversity of forest species associated with birch, oak, poplar, and willow. The surface area covered by trees expanded in the reserve since the 1980s (Kucharski et al., 2004), most probably resulting in the increased number of tree‐associated moths. Although it is also clearly visible that the succession of ruderal and agricultural flora results in a large number of common, often polyphagous eurytopic moths associated with common herbal plants. Further succession changes and drainage of Rąbień Bog will most probably result in shifts toward more opportunistic moth fauna. We also have to remember that moth caterpillars do not have a wide potential for changes in their food preferences. Therefore, it is impossible that larger groups of species were present in the bog before the changes in the flora, but were associated with different species of plants and only changed their diet after shifts in the floral communities. Species recorded in our study are mostly oligofagous, associated with relatively narrow group of ecologically and morphologically similar plants. Only a few species like *Arctia caja* might be treated as highly polyphagous and their diet cover not only a wide range of herbal plants but also various trees and shrubs (Buszko & Masłowski, 2012; Nowacki, 1998; Waring & Townsend, 2009). Some earlier studies showed that moth communities are good indicators of ecosystem condition (An & Choi, 2013). Our study demonstrated a low number of specialist taxa. This fact might indicate rather poor habitat quality. Based on a very limited data from other studies (Root et al., 2017; Tyler, 2020), we might assume that the restoration of flora might be the first and very important step for potential recolonization of Rąbień by rare or more specialized taxa, including at least some tyrphobionts and tyrphophiles. On the other hand, we must remember that Rabień is isolated from other larger forest complexes and peat bogs. This fact, along with the urbanization pressure, strong light pollution associated with urbanization might also affect moth populations (Kitching et al., 2000). Some studies showed that light pollution might affect migrations of moths (Boyes et al., 2020) and this factor is certainly important in the case of Rąbień. It might also explain the large number of typical herbal vegetation species, including many noctuids associated with ruderal plants (*Urtica*, *Plantago, Rumex, Taraxacum officinale*) that are normally rare or absent in forest ecosystems and peat bogs, but are very common in urbanized areas (Zhang et al., 2020) and might also expand in neighboring forests or peatbogs like Rąbień. It was already proven that light traps are an efficient tool for assessing local moth communities. as they attract the specimens from small distance (Truxa & Fiedler, 2012), therefore, our results show a reliable assessment of the community composition at investigated sites.

Moreover, urbanization might favor generalist species (Franzén et al., 2020) like polyphagous moths that were also present in our study. Therefore, we might expect an increase in those ecological groups of moths in the near future, since the development of new housing estates in the very close proximity to Rabień has continued in recent years and might influence further changes in vegetation of this area.

There is a great need for further and more extensive studies dedicated to plant and insect communities. We are dealing with a dynamic set of mutual interactions occurring at a very small area that is strongly affected by various types of disturbance. Without some further studies and management plans, this peat bog will completely degrade. At the same time, it still certainly deserves protection and can be treated as a good model system for studies of degradation processes and future restoration activities of peat bog communities.

## 5 CONCLUSIONS

In the 21st century, the urban wetlands face increasing degradation that started in the previous century, mostly due to peat extraction and irrigation. Currently, as they are often protected, like the Rąbień bog, other reasons become more important. Intentional destruction is replaced by processes that are side‐effects of settlement in the suburbs. The areas adjacent to the remnants of natural, protected landscapes are nowadays preferred for settlement over to downtown areas. That is why buffer zones of nature reserves face increasing pressure of estate development. This causes disturbances in water conditions, expansion of alien plants, and light pollution. As peatlands are strongly dependent to precipitation/evaporation conditions and air temperature, the problem of urban heat islands, along with the global warming, is accelerated by water level decrease, peat decomposition, and spontaneous afforestation. All these reasons cause the disappearance of tyrphophile and tyrphobiotic moth species which are replaced by taxa associated with common host tree species like birch and willows, especially in small bogs isolated in urban agglomerations. It raises a question, for how long Rąbień bog will keep its remnants of natural plant communities and give reasons for protection as a nature reserve. The degradation processes progress quickly and are inevitable. They also seem to be largely irreversible. Keeping high moth diversity in urban wetlands requires the necessary preventive management from local governments. A buffer zone with limited settlement, activities aimed at restoring the original water conditions, if possible, reduction of light pollution, and the physical limitation of the expansion of trees and shrubs should be obligatory elements of active forms of protection.

## AUTHOR CONTRIBUTIONS

**Mateusz Płóciennik:** Conceptualization (lead); data curation (lead); formal analysis (lead); investigation (lead); methodology (lead); project administration (lead); resources (lead); software (lead); supervision (lead); validation (lead); visualization (supporting); writing – original draft (lead). **Krzysztof Pabis:** Conceptualization (supporting); methodology (supporting); supervision (supporting); validation (supporting); writing – original draft (supporting). **Angelika Zima:** Conceptualization (supporting); investigation (supporting); resources (supporting); writing – original draft (supporting). **Leszek Kucharski:** Conceptualization (supporting); data curation (supporting); investigation (supporting); methodology (supporting); resources (supporting); validation (supporting); writing – original draft (supporting). **Robert Sobczyk:** Conceptualization (supporting); formal analysis (supporting); methodology (supporting); software (supporting); visualization (lead); writing – original draft (supporting).

## Supporting information


**Table S1:** Species occurrence at each site and habitats: (R ‐ present only on Różana, T ‐ present only on Torfowa, R/T ‐ present on both sampling sites). Note that abbreviations of habitats were used: PF ‐ pine forest, OW ‐ oak wood, HB ‐ hornbeam forest, ME ‐ meadows, RI ‐ riparian and alder forest, GA ‐ gardens, AB ‐ near buildings, AW ‐ around water, PA ‐ parks, FA ‐ farmlands, PB ‐ peat bogs.
**Table S2:** Species occurrence at each site and host plants of the collected moths taken from literature.

**Table S3:** Results of SIMPER analysis conducted for three groups of moths according to their host plants A, B, O.

**Table S4:** Moth taxa separated for A, B, O groups based on NMDS.

## Data Availability

The data that support the findings of this study are openly available in Dryad at http://doi.org/10.5061/dryad.xksn02vkx, reference number. They are also added to the paper as on‐line appendices (Table [Link], [Link], [Link]).
