# Supplementary material for: Plant–moth community relationships at the degraded urban peat‐bog in Central Europe
Source: Ecol Evol. 2023 Feb 13;13(2):e9808. doi: 10.1002/ece3.9808 (PMC9925946; doi:10.1002/ece3.9808)
Supplement: Supplementary file 2 — Table S3: Results of SIMPER analysis conducted for three groups of moths according to their host plants A, B, O. [file ECE3-13-e9808-s003.rtf]

Table S3. Results of SIMPER analysis conducted for three groups of moths according to their host plants A, B, O.    
SIMPER
Similarity Percentages - species contributions

Parameters

Transform: Presence/absence
Cut off for low contributions: 90,00%
Factor name: ABO

Group A

Average similarity: 30.84

Species  Av.Abund  Av.Sim  Sim/SD  Contrib%  Cum.%
Birch        0.53   11.17    0.60     36.22  36.22
Oak          0.41    7.27    0.42     23.56  59.78
Poplar       0.33    4.20    0.32     13.61  73.39
Willow       0.33    3.76    0.34     12.19  85.58
Alder        0.30    3.40    0.30     11.03  96.61

Group B

Average similarity: 12.52

Species              Av.Abund  Av.Sim  Sim/SD  Contrib%  Cum.%
 Sorrel                  0.25    2.37    0.25     18.92  18.92
Plantain                 0.25    2.37    0.25     18.92  37.84
Dandelion	             0.22    1.62    0.22     12.90  50.74
 Common nettle	       0.20    1.60    0.20     12.77  63.51
Grass                    0.17    1.46    0.15     11.69  75.20
Cleavers                 0.15    0.96    0.14      7.70  82.90
Pigweed                  0.12    0.56    0.11      4.48  87.38
Chickweed                0.12    0.49    0.11      3.87  91.26

Group O

Average similarity: 7.93

Species          Av.Abund  Av.Sim  Sim/SD  Contrib%  Cum.%
Scots pines	         0.23    2.64    0.22     33.33  33.33
Lichens              0.17    1.84    0.15     23.19  56.52
Spruce               0.20    1.72    0.19     21.74  78.26
Honeysuckle          0.10    0.54    0.08      6.76  85.02
 Campion             0.10    0.42    0.08      5.31  90.34

Groups A  &  B

Average dissimilarity = 98.61

                      Group A   Group B                                   
Species              Av.Abund  Av.Abund  Av.Diss  Diss/SD  Contrib%  Cum.%
Birch                    0.53      0.00    11.77     0.98     11.94  11.94
Oak                      0.41      0.02     9.68     0.77      9.81  21.75
Poplar                   0.33      0.00     7.29     0.65      7.39  29.14
Willow                   0.33      0.03     7.08     0.69      7.18  36.32
Adler                    0.30      0.03     6.84     0.64      6.93  43.25
 Sorrel                  0.01      0.25     5.58     0.56      5.66  48.91
Plantain                 0.00      0.25     5.45     0.56      5.53  54.44
 Common nettle           0.00      0.20     4.54     0.48      4.61  59.05
Dandelion                0.00      0.22     4.47     0.52      4.54  63.59
Grass                    0.00      0.17     4.41     0.42      4.47  68.06
Cleavers                 0.01      0.15     3.78     0.41      3.83  71.89
Pigweed                  0.00      0.12     2.80     0.35      2.84  74.73
Hornbeam                 0.11      0.02     2.66     0.36      2.70  77.43
Hawthorn                 0.10      0.00     2.60     0.31      2.63  80.07
Chickweed                0.00      0.12     2.57     0.36      2.61  82.67
Knotweed                 0.00      0.08     2.20     0.28      2.24  84.91
 Orach                   0.00      0.08     1.91     0.30      1.94  86.85
Clover                   0.00      0.07     1.73     0.26      1.75  88.60
Mountain Elm             0.07      0.00     1.55     0.27      1.57  90.17

Groups A  &  O

Average dissimilarity = 99.97

                        Group A   Group O                                   
Species                Av.Abund  Av.Abund  Av.Diss  Diss/SD  Contrib%  Cum.%
Birch                      0.53      0.00    13.82     0.99     13.83  13.83
Oak                        0.41      0.00    11.39     0.76     11.39  25.22
Poplar                     0.33      0.00     8.59     0.65      8.59  33.81
Willow                     0.33      0.00     7.89     0.68      7.89  41.70
Alder                      0.30      0.00     7.65     0.63      7.65  49.35
Scots pines                0.00      0.23     6.01     0.53      6.01  55.36
Lichens                    0.00      0.17     5.08     0.42      5.08  60.45
Spruce                     0.00      0.20     4.90     0.48      4.90  65.35
Hawthorn                   0.10      0.00     3.15     0.31      3.15  68.50
Honeysuckle                0.00      0.10     3.03     0.32      3.03  71.54
 Silene                    0.00      0.10     2.74     0.32      2.74  74.28
Common reed                0.00      0.10     2.74     0.32      2.74  77.02
Hornbeam                   0.11      0.00     2.71     0.34      2.71  79.74
Mountain Elm               0.07      0.00     1.81     0.27      1.81  81.54
 Impatiens         	   0.00      0.07     1.76     0.25      1.76  83.30
Medicam soapwort           0.00      0.07     1.63     0.26      1.63  84.94
Bulrush                    0.00      0.07     1.63     0.26      1.63  86.57
Fescue                     0.01      0.03     1.30     0.20      1.30  87.88
 

Groups B  &  O

Average dissimilarity = 99.91

                        Group B   Group O                                   
Species                Av.Abund  Av.Abund  Av.Diss  Diss/SD  Contrib%  Cum.%
 Sorrel                    0.25      0.00     6.46     0.56      6.47   6.47
Plantain                   0.25      0.00     6.46     0.56      6.47  12.93
Scots pines                0.00      0.23     6.10     0.53      6.10  19.03
Grass                      0.17      0.00     5.42     0.42      5.43  24.46
 Common nettle             0.20      0.00     5.42     0.48      5.42  29.89
Dandelion                  0.22      0.00     5.24     0.52      5.24  35.13
Lichens                    0.00      0.17     5.16     0.42      5.16  40.29
Spruce                     0.00      0.20     4.97     0.49      4.98  45.27
Bedstraw                   0.15      0.00     4.37     0.40      4.37  49.64
Pigweed                    0.12      0.00     3.38     0.35      3.38  53.02
Honeysuckle                0.00      0.10     3.08     0.32      3.08  56.11
Chickweed                  0.12      0.00     3.04     0.36      3.05  59.15
 Sticky                    0.00      0.10     2.78     0.32      2.78  61.94
Common reed                0.00      0.10     2.78     0.32      2.78  64.72
Knotweed                   0.08      0.00     2.71     0.29      2.71  67.44
 Orach                     0.08      0.00     2.27     0.30      2.28  69.71
Clover                     0.07      0.00     2.11     0.26      2.11  71.82
 Impatiens      	         0.00      0.07     1.78     0.25      1.79  73.61
Annual meadow grass        0.05      0.00     1.72     0.22      1.72  75.33
Medicam soapwort           0.00      0.07     1.66     0.26      1.66  76.99
Bulrush                    0.00      0.07     1.66     0.26      1.66  78.65
 Hawkbits                  0.02      0.03     1.59     0.22      1.59  80.24
Fescue                     0.02      0.03     1.30     0.22      1.30  81.54
 Bilberry                  0.05      0.00     1.27     0.23      1.27  82.81
 Dead-nettle               0.05      0.00     1.27     0.23      1.27  84.08
Yellow iris                0.02      0.03     1.19     0.22      1.19  85.28
Sisybium                   0.00      0.03     1.13     0.18      1.13  88.65
Raspberry                  0.00      0.03     1.13     0.18      1.13  89.78
Hedge bindweed             0.00      0.03     1.13     0.18      1.13  90.91
