# Supplementary material for: Plant–moth community relationships at the degraded urban peat‐bog in Central Europe
Source: Ecol Evol. 2023 Feb 13;13(2):e9808. doi: 10.1002/ece3.9808 (PMC9925946; doi:10.1002/ece3.9808)
Supplement: Supplementary file 3 — Table S4: Moth taxa separated for A, B, O groups based on NMDS. [file ECE3-13-e9808-s001.docx]

**Table S4.** Moth taxa separated for A, B, O groups based on NMDS.

| G**ROUP A** | | | | GROUP **B** | | | GROUP **O** | |
| --- | --- | --- | --- | --- | --- | --- | --- | --- |
| *Acronicta (Jochaera) alni*  *Acronicta (Acronicta) leporina*  *Acronicta (Triaena) psi*  *Acronicta (Viminia) rumicis*  *Agrochola (Sunira) circellaris*  *Allophyes (Allophyes) oxyacanthae*  *Bena bicolorana*  *Ceramica pisi*  *Colocasia coryli*  *Conistra (Conistra) rubiginosa*  *Conistra (Conistra) vaccinii*  *Cosmia (Calymnia) trapezina*  *Euplexia lucipara*  *Eupsilia transversa*  *Ipimorpha subtusa*  *Lacanobia (Dianobia) thalassina*  *Lithophane (Lithophane) furcifera*  *Lithophane (Lithophane) ornitopus*  *Orthosia (Monima) cerasi*  *Orthosia (Semiophora) gothica*  *Orthosia (Orthosia) incer*  *Anorthoa munda* | *Polia (Polia) nebulosa*  *Tholera cespitis*  *Acasis viretata*  *Abraxas (Calospilos) sylvata*  *Angerona prunaria*  *Apocheima hispidaria*  *Apocheima pilosaria*  *Biston betularia*  *Biston strataria*  *Cabera pusaria*  *Campaea margaritata*  *Colotois pennaria*  *Comibaena bajularia*  *Crocallis elinguaria*  *Cyclophora (Codonia) punctaria*  *Ectropis crepuscularia*  *Electrophaes corylata*  *Ennomos alniaria*  *Ennomos (Deuteronomos) erosaria*  *Epirrita autumnata*  *Geometra papilionaria*  *Hemithea aestivaria* | *Hypomecis punctinalis*  *Hypomecis roboraria*  *Lobophora halterata*  *Lomaspilis marginata*  *Lomographa temerata*  *Macaria alternata*  *Macaria notata*  *Phigalia pilosaria*  *Plagodis dolabraria*  *Selenia dentaria*  *Selenia tetralunaria*  *Calliteara pudibunda*  *Euproctis (Sphrageidus) similis*  *Lymantria (Portheria) dispar*  *Clostera anastomosis*  *Drymonia querna*  *Drymonia ruficornis*  *Furcula furcula*  *Notodonta ziczac*  *Peridea anceps*  *Phalera bucephala*  *Pheosia tremula* | *Arctia caja*  *Achlya flavicornis*  *Drepana curvatula*  *Drepana falcataria*  *Falcaria lacertinaria*  *Habrosyne pyritoides*  *Ochropacha duplaris*  *Tetheella fluctuosa*  *Watsonalla binaria*  *Odonestis pruni*  *Apoda limacodes*  *Laothoe populi*  *Smerinthus ocellata*  *Pseudoips prasinana*  *Polia (Polia) bombycina*  *Hydrelia flammeolaria*  *Ptilodon capucina* | *Abrostola triplasia*  *Agrotis (Agrotis) exclamationis*  *Agrotis (Agrotis) segetum*  *Agrotis (Agrotis) vestigialis*  *Amphipyra (Amphipyra) livida*  *Anarta (Calocestra) trifolii*  *Autographa gamma*  *Axylia putris*  *Calamia tridens*  *Charanyca (Rusina) ferruginea*  *Charanyca (Charanyca) trigrammica*  *Deltote bankiana*  *Denticucullus pygmina*  *Diachrysia chrysitis*  *Diarsia brunnea*  *Diarsia florida*  *Dypterygia scabriuscula*  *Hada plebeja*  *Hoplodrina ambigua*  *Hoplodrina blanda*  *Hoplodrina octogenaria* | *Hypena (Hypena) proboscidalis*  *Lacanobia (Diataraxia) oleracea*  *Luperina testacea*  *Macdunnoughia (Macdunnoughia) confusa*  *Mamestra brassicae*  *Melanchra persicariae*  *Mesapamea secalis*  *Mniotype satura*  *Mythimna (Mythimna) conigera*  *Mythimna (Hyphilare) ferrago*  *Noctua pronuba*  *Oligia strigilis*  *Pseudeustrotia candidula*  *Rhizedra lutosa*  *Trachea atriplicis*  *Xestia (Megasema) c-nigrum*  *Xestia (Xestia) triangulum*  *Xylena (Xylena) vetusta*  *Charanyca (Rusina) ferruginea*  *Chiasmia clathrata*  *Epirrhoe alternata* | *Pennithera firmata*  *Perizoma alchemillata*  *Pseudeustrotia candidula*  *Timandra comae*  *Xanthorhoe ferrugata*  *Xanthorhoe montanata*  *Xanthorhoe spadicearia*  *Stauropus fagi*  *Phragmatobia fuliginosa*  *Spilosoma lubricipeda*  *Spilarctia (Spilarctia) lutea*  *Deilephila elpenor*  *Patania ruralis*  *Hydraecia micacea*  *Idaea aversata* | *Globia algae*  *Deltote pygarga*  *Acontia (Emmelia) trabealis*  *Lacanobia (Dianobia) contigua*  *Laspeyria flexula*  *Leucania obsoleta*  *Mythimna (Mythimna) pallens*  *Nonagria typhae*  *Panolis flammea*  *Sideridis (Aneda) rivularis*  *Cepphis advenaria*  *Ecliptopera silaceata*  *Eupithecia venosata*  *Eupithecia indigata*  *Hylaea fasciaria*  *Macaria brunneata*  *Macaria liturata*  *Xanthorhoe biriviata*  *Xanthorhoe fluctuata*  *Lymantria (Lymantria) monacha*  *Eilema (Manulea) complana* | *Eilema (Wittia) sororcula*  *Miltochrista miniata*  *Pelosia muscerda*  *Thyatira batis*  *Dendrolimus pini*  *Euthrix potatoria*  *Sphinx pinastri* |
